# Two-hierarchical nonnegative matrix factorization distinguishing the fluorescent targets from autofluorescence for fluorescence imaging

**DOI:** 10.1186/s12938-015-0107-4

**Published:** 2015-12-15

**Authors:** Shaosen Huang, Yong Zhao, Binjie Qin

**Affiliations:** School of Biomedical Engineering, Shanghai Jiao Tong University, Dongchuan Road, Shanghai, China

**Keywords:** Multispectral fluorescence imaging, Equality constraint, Spectral unmixing, Autofluorescence, Nonnegative matrix factorization, Two-hierarchical

## Abstract

**Background:**

Nonnegative matrix factorization (NMF) has been used in blind fluorescence unmixing for multispectral in-vivo fluorescence imaging, which decomposes a mixed source data into a set of constituent fluorescence spectra and corresponding concentrations. However, most classical NMF algorithms have ill convergence problems and they always fail to unmix multiple fluorescent targets from background autofluorescence for the sparse acquisition of multispectral fluorescence imaging, which introduces incomplete measurements and severe discontinuities in multispectral fluorescence emissions across the multiple spectral bands.

**Methods:**

Observing the spatial distinction between the diffusive autofluorescence and the sparse fluorescent targets, we propose to separate the mixed sparse multispectral data into equality constrained two-hierarchical updating within NMF framework by dividing the concentration matrix of entire endmembers into two hierarchies: the fluorescence targets and the background autofluorescence. Specifically, when updating concentrations of multiple fluorescent targets in the two-hierarchical NMF, we assume that the concentration of autofluorescence is fixed and known, and vice versa. Furthermore, a sparsity constraint is imposed on the concentration matrix components of fluorescence targets only.

**Results:**

Synthetic data sets, in vivo fluorescence imaging data are employed to demonstrate and validate the performance of our approach. The proposed algorithm can achieve more satisfying results of spectral unmixing and autofluorescence removal compared to other state-of-the-art methods, especially for the sparse multispectral fluorescence imaging.

**Conclusions:**

The proposed algorithm can successfully tackle the sparse acquisition and ill-posed problems in the NMF-based fluorescence unmixing through equality constraint along with partial sparsity constraint during two-hierarchical NMF optimization, at which fixing sparsity constrained target fluorescence can make the update of autofluorescence as accurate as possible and vice versa.

## Background

Due to Shimomura, Chalfie and Tsien’s shared discovery and development of the green fluorescent protein [1], noninvasive in-vivo fluorescence imaging has become a powerful technique at labeling and tracking cells in tissues for biological and medical sciences, such as drug discovery and disease diagnosis [2]. However, the overlap of multiple fluorescence spectra introduces the cross-talk between multiple fluorescent targets in multispectral fluorescence imaging. Especially, when excited in the visible to near-infrared part of the electromagnetic spectrum, some natural fluorescent molecules in tissues and food (such as collagens, lipofuscin, and melanin) can also produce fluorescence photons called autofluorescence (AF) [2–5], which spreads everywhere in tissues with typically containing other non-sparse instrument-based background noise (such as dark current, residual bulk image in CCD, and leakage light from exciting filters). Therefore, the strong spatial and spectral overlap between different target fluorophores and background AF makes it very difficult to unmix multiple fluorescent targets, complicating the analysis of multiple fluorophores in multispectral fluorescence imaging.

To solve the overlapping problem in fluorescence imaging, spectral unmixing (SUM) is used to decompose the mixed spectra data $\mathbf {D}$ into a product of pure spectral signatures $\mathbf {S}$, i.e., endmembers, and corresponding concentrations $\mathbf {C}$ (or abundances, mixing weights), representing the contribution to the observed fluorescence radiance from the corresponding endmember. Before we dig into some fluorescence unmixing methods, it will help us to have a general understanding of the following observations in fluorescence imaging: first, due to in-vivo fluorescence imaging measuring the diffuse radiance from the surface of scattering animal tissue after the fluorescent light transport in tissues, a fluorophore inside animal tissue might make a significant contribution to a cloud of neighboring pixels instead of only the pixel geometrically associated with it, such that the quantity $\mathbf {C}$ for multiple fluorophores represents the signal distribution of these multiple fluorophores instead of the concentrations of fluorophores in the pixel [3]; second, the emitted fluorescent light travel in diffusive tissues can result in the decreasing intensity and spectral distortions. However, the spectrum distortion of signatures $\mathbf {S}$ is insignificant in front of intensity loss of deep embedded fluorophores [6]. Therefore, the signatures of multiple diffusive fluorescent sources are relatively stable in contributing to the mixed spectra data in fluorescence imaging; third, as the fluorescence signal attenuates exponentially with the distance light travels, a background AF signal remains constant and becomes a serious limiting factor. The analysis of a fluorescence signal polluted by the background AF may lead to a wrong localization of the target fluorophore. In the light of the above mentioned observations, the spectral unmixing is therefore precisely referred to as a blind source separation approach, which is a necessary preprocessing step to remove unwanted AF and unmix different spectra of interest. By returning separated pure fluorescence contribution data, the SUM method not only gives contrast-enhanced [6] signal distribution of each target fluorophore in 2D planar imaging to enable comparing fluorescence sources at similar imaging conditions and comparable depth, but also presents the possibility to perform accurate fluorescence tomographic reconstructions confirming the 3D position of marked samples.

According to different endmember estimation methods, SUM has been classified as either supervised [3, 7] or unsupervised. In practice, a user-selected region containing a single fluorescent target or a spectral library for multiple known fluorescent targets present in the sample is chosen to provide the endmember information when implementing supervised SUM. However, it is difficult to select an appropriate region solely containing single fluorescence or to make extensive calibration in the spectral library acquisition ensuring the accuracy of the unmixing algorithm. Therefore, various unsupervised SUM methods [8, 9] (also called blind source separation) have been developed to estimate spectra and concentrations simultaneously without exact *priori* knowledge about constituent spectra.

To blindly apply spectral unmixing for fluorescence imaging, nonnegative matrix factorization (NMF) [10, 11] has been used to find the matrices $\mathbf {S}$ and $\mathbf {C}$ by minimizing the cost function *F* representable as a difference between $\mathbf {D}$ and $\mathbf {CS}$ in fluorescence spectroscopy [4, 12–15]. However, due to the cost function of NMF being not convex in the search spaces of $\mathbf {S}$ and $\mathbf {C}$ simultaneously, starting from different initial search points results in different values for the elements of $\mathbf {S}$ and $\mathbf {C}$. Therefore, the NMF suffers form the ill convergence problem and the final solution is easily caught in the local minimum of the cost function. Especially for the sparse multispectral fluorescence imaging data that are acquired in a relatively small number (3–10) of spectral bands, the ill convergence problem is easily exacerbated due to the sample deficiency and severe discontinuity of multispectral fluorescence emissions, though this sparse imaging can be fast and cost-effective in clinical applications. In multispectral fluorescence imaging, the multiple-target fluorophores with their unimodal spectrum emission peaks are sparse sources in both the spatial and the spectral domains. Such source sparsity is widely used as a regularized constraint in fluorescence unmixing studies [4, 12–14, 16], among which a few studies were interested in the sparsity of matrix $\mathbf {S}$. However, unlike the hyperspectral imaging [17, 18], the sparse acquisition of multispectral fluorescence imaging is inevitable to cause spectrum leakage and fence effect in the spectrum of fluorescence target such that the rows of $\mathbf {S}$ are not true expressions of spectrally sparse fluorescence emissions. Furthermore, Montcuquet et al. [4] reported that, due to the currently used fluorescent markers traveling in blood and lymphatic canals and always spreading so-called nonspecific signal everywhere in tissues, the spatial sparsity constraints alone may seem inappropriate for the unmixing methods to detect the fluorescent source signal from the nonspecific signal. Moreover-and to the best of our knowledge-NMF methods fail in discriminating the spatial sparsity of the multiple-target fluorescence signal and the spatial non-sparsity of the AF (including the nonspecific signal).

Considering the above-mentioned factors, we propose a two-hierarchical NMF (thNMF) procedure to distinguish the updating of matrix components for the multiple fluorescent targets from the background AF. Our proposed two-hierarchical NMF is first inspired by the equality constraint in alternating least squares procedures [19], where the known columns of $\mathbf {C}$ are fixed during the update process and the unknown columns of $\mathbf {C}$ are updated with the sub-cost function which has already removed the part of known columns of $\mathbf {C}$ and the corresponding rows of $\mathbf {S}$. To some degree, equality constrained least squares algorithm can be regarded as separating one known hierarchy from one unknown hierarchy during the update process although the known hierarchy is fixed within hierarchical alternating least squares (HALS) algorithm [20–22].

By exploiting the difference between the spatial distributions of target fluorophores and background AF within the equality constrained HALS framework, we propose thNMF with the following two main contributions: first, rather than updating the whole matrix $\mathbf {C}$ (and corresponding $\mathbf {S}$) in minimizing the cost function *F*, the thNMF treats the diffusive AF separately from the sparse fluorescent targets and divides the whole minimization process into two different sub-processes, which fix the known column of matrix $\mathbf {C}$ for background AF (resp. target fluorophores) during the updating unknown columns of $\mathbf {C}$ for target fluorophores (resp. background AF) with the sub-cost function having removed the part of known columns of $\mathbf {C}$ and the corresponding rows of $\mathbf {S}$, whereby we can prevent thMNF from mixing these two different components in NMF decomposition; second, the two-hierarchical scheme can potentially facilitate imposing different appropriate constraints on the target and background components in the two sub-processes to obtain desired unmixing results. In this paper, according to the spatial sparsity difference between the target fluorophores and background AF, we solely impose the partial sparsity constraint on the fluorescent targets to accurately separate the sparse multi-target fluorophores and non-sparse background AF. The remainder of this paper is organized as follows. Section II describes the materials and methods of proposed thNMF algorithm. Section III provides experimental results on synthetic and in-vivo fluorescence imaging data. A discussion and conclusions are given in section IV.

## Methods

### Two-hierarchical NMF

Most existing spectral unmixing approaches are based on linear mixture model, which can be expressed as

1 $$\begin{aligned} \mathbf {D=CS+E} \end{aligned}$$

where matrix $\mathbf {D}\in \mathbb {R}_{+}^{N\times L}$ describes multispectral fluorescence imaging data where each column corresponds to a single fluorescent image of a special spectral band. *N* is the number of total pixels in a single image and *L* is the number of acquisition spectral bands. $\mathbf {C} = [{\mathbf {c}_1},{\mathbf {c}_2}, \ldots ,{\mathbf {c}_k}] \in {\mathbb {R}_+^{N \times K}}$ is called concentration matrix where each column ${\mathbf {c}_i}$ represents the concentration distribution of a special endmember and $\mathbf {S} = {[{\mathbf {s}_1},{\mathbf {s}_2}, \ldots ,{\mathbf {s}_k}]^T} \in {\mathbb {R}_+^{K \times L}}$ is the endmember spectrum matrix where $\mathbf {s}_i$ is a vector corresponding to the emission spectrum of the *i*th endmember. *K* denotes the number of endmembers. $\mathbf {E}$ is the related noise matrix.

The aim of NMF methods is to find the nonnegative best solution couple ($\mathbf {C,S}$) whose product $\mathbf {CS}$ best approaches the original data matrix $\mathbf {D}$. To solve the NMF problem, an alternating nonnegative least squares (ANLS) algorithm [22, 23] regards it as a convex optimization subproblem to find the optimal factor $\mathbf {C}$ corresponding to a fixed factor $\mathbf {S}$ and vice versa. When partitioning variables into one single variable at a time in each convex subproblem in the framework of ANLS, we can get a simple univariate quadratic problem, such as ${\mathbf {C}_{ik}} \leftarrow \text {argmin}{_{{\mathbf {C}_{ik}} \ge 0}}\left\| {\mathbf {D-CS}} \right\| _2^2$, which admits a closed-form solution. Moreover, since the optimal value for a given entry of $\mathbf {C}$ does not depend on the other entries of the same column, one can optimize alternatively whole columns of $\mathbf {C}$, with

2 $$\begin{aligned} \begin{array}{l} {\mathbf {C}_{:k}} \leftarrow \text {argmin}{_{{\mathbf {C}_{:k}} \ge 0}}\left\| {\mathbf {D-CS}} \right\| _2^2 \\ \;\;\;\;\;\;\;= \text {argmin}{_{{\mathbf {C}_{:k}} \ge 0}}\left\| {{\mathbf {R}_k} - {\mathbf {C}_{:k}}{S_{k:}}} \right\| _2^2 \\ \end{array} \end{aligned}$$

3 $$\begin{aligned} \begin{array}{l} {\mathbf {S}_{k:}} \leftarrow \text {argmin}{_{{\mathbf {S}_{k:}} \ge 0}}\left\| {\mathbf {D-CS}} \right\| _2^2 \\ \;\;\;\;\;\; = \text {argmin}{_{{\mathbf {S}_{k:}} \ge 0}}\left\| {{\mathbf {R}_k} - {\mathbf {C}_{:k}}{\mathbf {S}_{k:}}} \right\| _2^2 \\ \end{array} \end{aligned}$$

where ${\mathbf {R}_k} = \mathbf {D} - \sum \nolimits _{i \ne k} {{\mathbf {C}_{:i}}{\mathbf {S}_{i:}}}$ is the *k*th residual matrix. This new method is referred to as HALS [20–22]. Under the framework of HALS algorithm, it seems convenient to add different constraints to each column of $\mathbf {C}$ or each row of $\mathbf {S}$. HALS algorithms with a sparsity constraint [25] and a constraint for maximum spatial dispersion [26] have been applied in the area of hyperspectral unmixing.

Actually, in the HALS computation framework, the multiple fluorescent targets and background AF are amenable to being treated as different hierarchies due to their having fundamentally different characteristics in the spatial and spectral distributions. Firstly, the fluorescent targets are locally accumulated in specific biological tissues and have sparse distributions in the spatial areas, while background AF propagates at all directions and diffuses uniformly over large spatial areas. Secondly, the spectrum of a single fluorescent target is narrow and only has one emission peak in spectral excitation and emission, while the background AF widely spreads at all visible light range and has a slowly varying spectral distribution. However, this spectrum distinction between fluorescence target and background AF is substantially weakened in the sparse multispectral fluorescence imaging, which introduces spectrum leakages between neighboring spectral bands and severe discontinuities of spectral emissions for both fluorescence targets and background AF. Therefore, it is reasonable for NMF to emphasize on the continuous characteristics of the spatial distributions for fluorescent targets and background AF and only introduce appropriate regularization constraint into the concentration matrix $\mathbf {C}$ in this work.

As for exploiting the different spatial regularizations for the fluorescent targets and background AF, we divide the whole update of concentration matrix $\mathbf {C}$ into two different but relevant subproblems for the fluorescent targets and background AF. Inspired by the equality constraints on variables during alternating least squares procedures [19] and the HALS, we propose two-hierarchical NMF for the sparse multispectral fluorescence imaging. Assuming that the original cost function with an appropriate sparsity constraint $J(\mathbf {C})$ is $F\left( \mathbf {C},\mathbf {S} \right) =\left\| \mathbf {D}-\mathbf {CS} \right\| _{2}^{2}+\lambda J\left( \mathbf {C} \right)$ , where $\lambda \in {{\mathbb {R}}^{+}}$ is a scalar that weighs the contribution of the sparsity measure function $J(\mathbf {C})$ of the matrix $\mathbf {C}$, which is usually regarded as a regularization term. According to the ANLS algorithm, we can obtain the update rules

4 $$\begin{aligned} \mathbf {S} \leftarrow \text {argmin}{_{\mathbf {S} \ge 0}}\mathrm{{}}\left\| {\mathbf {D-CS}} \right\| _2^2 \end{aligned}$$

5 $$\begin{aligned} \mathbf {C} \leftarrow \text {argmin}{_{\mathbf {C} \ge 0}}\mathrm{{}}\left\| {\mathbf {D-CS}} \right\| _2^2 + \lambda J\mathrm{{(}}\mathbf {C}\mathrm{{)}} \end{aligned}$$

Considering the concentration distinction between fluorescent targets and background AF, we let ${\mathbf {C}=[\mathbf {C}_F\ \mathbf {C}_A]}$ and $${\mathbf {S} = [\begin{array}{l} {{\mathbf {S}_F}} \\ {{\mathbf {S}_A}} \\ \end{array}]}$$. Then the problem of Eq. (5) can be divided into two subproblems:

6 $$\begin{aligned} {\mathbf {C}_F} \leftarrow \text {argmin}{_{{\mathbf {C}_F} \ge 0}}\left\| {{\mathbf {D}_F} - {\mathbf {C}_F}{\mathbf {S}_F}} \right\| _2^2 + {\alpha }{J_1}\left( {{\mathbf {C}_F}} \right) \end{aligned}$$

7 $$\begin{aligned} {\mathbf {C}_A} \leftarrow \text {argmin}{_{{\mathbf {C}_A} \ge 0}}\left\| {{\mathbf {D}_A} - {\mathbf {C}_A}{\mathbf {S}_A}} \right\| _2^2 + {\beta }{J_2}\left( {{\mathbf {C}_A}} \right) \end{aligned}$$

where ${\mathbf {C}_F}$ and ${\mathbf {S}_F}$ denote the concentrations and fluorescence spectra of fluorescent targets, ${\mathbf {C}_A}$ and ${\mathbf {S}_A}$ denote the concentration and spectrum of background AF. ${\mathbf {D}_F} = \mathbf {D}-{\mathbf {C}_A}{\mathbf {S}_A}$ and $\mathbf {D_A} = \mathbf {D}-{\mathbf {C}_F}{\mathbf {S}_F}$ represent the residual matrix. $\alpha \in {{\mathbb {R}}^{+}}$ and $\beta \in {{\mathbb {R}}^{+}}$ are scalars which weigh the contributions of the spatial regularization functions $J(\mathbf {C}_F)$ of the matrix $\mathbf {C}_F$ and $J(\mathbf {C}_A)$ of the matrix $\mathbf {C}_A$, respectively. In the real experiment, we assume that only one endmember is enough to represent background AF which includes all noise and other background signals, so that it means that $\mathbf {C}_F \in \mathbb {R}_ + ^{N \times (K - 1)}$, ${\mathbf {S}_F} \in \mathbb {R}_ + ^{(K - 1) \times L}$, ${\mathbf {C}_A} \in \mathbb {R}_ + ^{N \times 1}$ and ${\mathbf {S}_A} \in \mathbb {R}_ + ^{1 \times L}$.

Because the update rule of HALS is the same as the classical multiplicative rule for NMF although the solution may be different. We can use the multiplicative update rule to optimize the problems of Eqs. (4) and (5) at last. Besides, many constraints can be easily introduced into NMF based on the scheme of multiplicative update rules, such that we can simply apply the appropriate constraints to optimize the solutions of ${\mathbf {C}_F}$ and ${\mathbf {C}_A}$.

Besides the convenience of introducing appropriate constraints to different hierarchies of endmembers, there is another important reason why we propose the two-hierarchical structures for the solutions to subproblems of the Eq. (5). Although there are some classical NMF algorithms which can only add appropriate constraints to some specific endmembers, they don’t change the update rules of basic NMF framework but add constraints into some specific endmembers after the update of whole $\mathbf {C}$. In fact, updating whole $\mathbf {C}$ cannot prevent the classical NMF from mixing the target fluorophores and background AF in the real experiment. However, the hierarchical updating of Eqs. (6) and (7) for optimizing Eq. (5) is fully different from the updating framework of classical NMF, its advantage can be explained with the equality constraints in the work of Van Benthem et al. [19]. When updating the concentrations of multiple fluorescent targets ${\mathbf {C}_F}$, we assume that the concentration of AF ${\mathbf {C}_A}$ is fixed and known, and vice versa. In an iterative process of updating $\mathbf {C}$, it seems that this assumption is a special “equality constraint” for the solution to ${\mathbf {C}_F}$ (or ${\mathbf {C}_A}$). The equality constrained optimization is used to fix some components and to free other ones, such that it not only enables us to avoid unexpected components in the concentration matrix but also helps us gets more precise information on unknown concentrations. With an ongoing iterative process of hierarchical updating, we can obtain the desired concentrations of ${\mathbf {C}_F}$ and ${\mathbf {C}_A}$ according to different appropriate constraints. Furthermore, a more accurate ${\mathbf {C}_F}$ (or ${\mathbf {C}_A}$) will lead to a more accurate ${\mathbf {C}_A}$ (or ${\mathbf {C}_F}$) based on the hierarchical updating.

### Constraints

Based on the basic framework of thNMF, different appropriate constraints can be imposed separately on each hierarchy of multiple fluorescent targets and background AF in in-vivo fluorescence imaging. As mentioned in the introduction section, a spatial sparsity constraint is appropriate for concentrations of multiple fluorescent targets with its improving the uniqueness of the decomposition along with enforcing a local-based representation. There are several popular ways to impose sparsity constraints on NMF. In Hoyer’s non-negative sparse coding [27] framework, Frobenius norm is combined with ${{l}_{1}}$-norm of matrix $\mathbf {C}$ or $\mathbf {S}$ (the sparseness penalty term) to construct a cost function. Hoyer’s second method [28] has enforced a desired sparsity degree by means of nonlinear projection at each NMF iteration based on a sparsity measure from the relationship between ${{l}_{1}}$ and ${{l}_{2}}$-norms. With concentration matrix’s *k*th column being a nonzero *N* dimensional vector, the sparsity measure of $\mathbf {C}_k$ is defined as

8 $$\begin{aligned} \phi (\mathbf {C}_k)=\frac{\sqrt{N}-\left( {\sum \nolimits _{n=1}^{N}{\left| {{\mathbf {C}}_{nk}} \right| }}/{\sqrt{\sum \nolimits _{n=1}^{N}{\mathbf {C}_{nk}^{2}}}}\; \right) }{\sqrt{N}-1}\ \in \left[ 0,1 \right] \end{aligned}$$

The first technique is conceptually simpler than the second technique but requires determining an appropriate penalty parameter $\lambda$ which has a wide range of parameter values, while the second technique is more complicated but allows user to choose a priori sparsity value that ranges from 0 for non-sparse result to 1 for extremely sparse result. Being different from Hoyer’s second method enforcing the solution of matrix $\mathbf {C}$ to reach a fixed sparsity value, Montcuquet et al. proposed a thresholding step in each updating iteration to help the algorithm converge to a more accurate solution [4].

For the framework of thNMF, it is easy to combine with different sparsity constraints mentioned above. For the simplicity and speed of iteration, we choose the ${{l}_{1}}$-norm as the sparsity measure function in the Eq. (6) to enforce the concentrations’ sparseness for multiple fluorescent targets, i.e., the Eq. (6) for the thNMF algorithm (see Algorithm 1) can be modified as

9 $$\begin{aligned} {\mathbf {C}_F} \leftarrow \text {argmin}{_{{\mathbf {C}_F} \ge 0}}\left\| {{\mathbf {D}_F} - {\mathbf {C}_F}{\mathbf {S}_F}} \right\| _2^2 + {\alpha }\mathrm{{}}\left\| {\mathbf {C}_F} \right\| _1 \end{aligned}$$

In the hierarchy of fluorescent targets, The updating rule of $\mathbf {C}_F$ is similar to the method first proposed by Hoyer [27]:

10 $$\begin{aligned} \mathbf {C}_F \leftarrow {\mathbf {C}_F}.\mathrm{{*}}\frac{{\left[ {{\mathbf {C}_F}\mathbf {S}_F^T} \right] }}{{\left[ {{\mathbf {C}_F}{\mathbf {S}_F}\mathbf {S}_F^T + {\alpha }} \right] }} \end{aligned}$$

The ${\alpha }$ is a small regularization parameter that balances the tradeoff between the approximation error and constraints. Compared with the sparsity value $\phi$, the penalty parameter $\alpha$ for ${{l}_{1}}$-norm is difficult to control. For the convenience of determining the penalty parameter and quantitative analysis of the sparsity degree among different algorithms, we use the similar method of [24] to adaptively control the $\alpha$: $\alpha$ for $\mathbf {C}_F$ is initialized to 0.01, and after each iteration, $\alpha$ is increased by 5 percent if average sparsity of $\mathbf {C}_F$ is below the target sparsity $\phi$, and is decreased by 5 percent otherwise. The desired sparsity value $\phi$ is set to 0.65–0.85 for in-vivo fluorescence imaging. In the real application, it may occur that the $\alpha$ is fluctuating during the iteration. To deal with this phenomenon, if the parameter $\alpha$ fluctuates during the adjacent 20 iterations, the adaptive procedure of $\alpha$ is terminated and $\alpha$ becomes constant.

For the simplicity and overall performance of spectral unmixing, we only choose the appropriate constraint on the fluorescent targets rather than on the background AF though it is easy to introduce an appropriate constraint on the AF in Eq. (7).

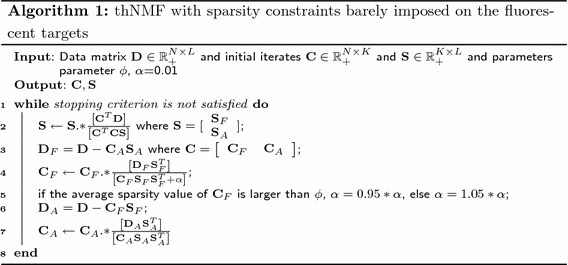



During the iterative process, the entries of $\mathbf {D}_F$ and $\mathbf {D}_A$ are set to 0 if they are less than 0. At *j*th iteration, the value of cost function $F{(\mathbf {C,S})^j}$ will be computed and the algorithm will be stopped if $\left| {F{{(\mathbf {C,S})}^j} - F{{(\mathbf {C,S})}^{j - 1}}} \right| < tol$, which is chosen equal to 0.0001.

### Initialization of $\mathbf {C}$ and $\mathbf {S}$

Due to NMF suffering from the ill-posed problem, the final decomposition solution strongly depends on NMF’s initialization. Moreover, for the NMF with different sparsity constraints on the different endmemebers, it is important to have relatively accurate initial estimates because specific endmembers correspond to specific sparsity degrees for the concentrations $\mathbf {C}$. Some authors initialize $\mathbf {S}$ using asymmetric Gaussian functions [14] or exponential initialization matrix [12] to simulate the quintessential fluorochrome spectra, while other works [15] exploit the statistical characteristics of the source spectra signals for the initialization of $\mathbf {S}$. By adopting similar methods in the fluorescence imaging [4] and hyperspectral unmixing [29], we use calibration spectra to initialize the signature matrix $\mathbf {S}$. The calibration spectra of multiple target fluorescence (fluorescent dyes) are acquired at the multiple emission filters by pre-calibration ex-vivo experiments in the same imaging conditions as the following in-vivo experiments, while the AF spectrum is the average spectrum acquired in some chosen regions of mouse with no fluorescent dyes. The concentration matrix $\mathbf {C}$ is obtained by keeping spectra $\mathbf {S}$ fixed during the first 10 iterations with the classical multiplicative update for NMF.

## Results

### Experimental results with synthetic data

In this section, we define a simulated experiment to quantitatively evaluate the performance of thNMF algorithm compared to other four classic spectral unmixing algorithms: multivariate curve resolution-alternating least squares (MCR-ALS) [3], HALS-based NMF with ${{l}_{1}}$-norm constraint on $\mathbf {C}$ (NMF${{l}_{1}}$) [24], NMF with sparsity constraints on $\mathbf {C}$ (NMFsc) [28], sparse NMF (sNMF) [4]. MCR-ALS has been used in the chemometrics of biological tissues [17] and small animal fluorescence imaging system [3], while NMF${{l}_{1}}$, NMFsc and sNMF are different NMF algorithms with different sparsity constraints. According to the above-mentioned initializations for $\mathbf {C}$ and $\mathbf {S}$, we use the same initial calibration spectra [4] and concentration matrix estimates for all NMF-based algorithms in all following experiments.

**Fig. 1:**
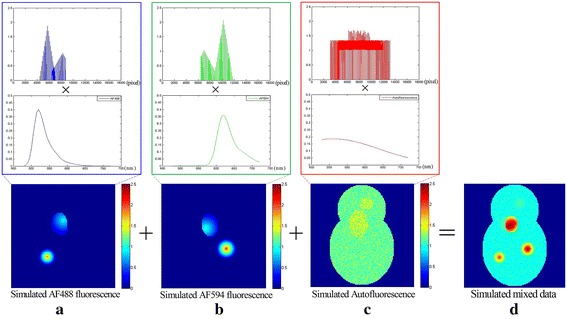
Synthetic data. The three rows are concentration vectors, fluorescence spectra and data spatial distributions. The first three columns correspond to the three different endmembers. **a** AF488. **b** AF594, **c** AF. **d** Simulated phantom acquired at 555 nm

To test the proposed method’s performance on normal multispectral image data, we build a typical phantom [4] that is composed of two specific fluorescence objects and one strong AF object. The fluorescence spectra of specific fluorescence parts are fitted curves of Alexa Fluor (AF) 488 (see Fig. 1a) and 594 (see Fig. 1b) fluorescence dyes at their spectrum wavelength from 488 to 720 nm with interval of 5 nm, while the fluorescence spectrum of AF is a relatively slow-varying curve in the same range (see Fig. 1c). The concentration vectors of AF488 and AF594 consist of two parts: one part is pure fluorescent dye and other part is mixed with AF488 and AF594 (see Fig. 1a, b). The simulated fluorescent target signal (or AF signal) is a product of the concentration vector and the corresponding fluorescence spectrum. We can see that AF’s intensity in Fig. 1a, c has the same order of magnitude as that for the fluorescent target signal. Finally, a simulated phantom is obtained by adding the two specific fluorescent signals and the AF signal together (see Fig. 1d). At last, we provide a sensitivity study with the simulated data that are added with different zero-mean white Gaussian noises with different signal-to-noise ratios (SNRs).

We use spectral angle distance (SAD) [30] to evaluate the endmember spectrum estimation, and mean square error (RMSE) [31] to evaluate the concentration estimation. These two criteria are used to measure the similarity between the unmixed result and its standard (or ground truth) value. In general, the smaller value of the SAD (or RMSE) means the better similarity and the better unmixing performance. For the *k*th endmember, the SAD is defined as

11 $$\begin{aligned} \text {SAD}\left( {\varvec{{\mathrm{{\hat s}}}}},\mathbf {s} \right) ={{\cos }^{-1}}\left( \frac{{{{\varvec{{\mathrm{s}}}}}^{T}}{\varvec{{\hat{\mathrm{s}}}}}}{\sqrt{{{\mathbf {s}}^{T}}\mathbf {s}}\sqrt{{{{{\varvec{{\hat{\mathrm{s}}}}}}}^{T}}{\varvec{{\hat{s}}}}}} \right) \end{aligned}$$

where $\mathbf {s}$ is the standard spectral vector and $\hat{\mathbf {s}}$ its estimate. RMSE is defined as

12 $$\begin{aligned} \text {RMS}{{\text {E}}_{k}}=\sqrt{\frac{1}{N}\sum \limits _{i=1}^{N}{{{\left( {{\mathbf {C}}_{ki}}-{{{{\varvec{{\hat{C}}}}}}_{ki}} \right) }^{2}}}} \end{aligned}$$

where $\mathbf {C}_{ki}$ and ${{\hat {\mathbf{C}}}_{ki}}$ are the standard and the estimated concentration of a specific pixel, respectively. At last, the average value of all endmembers’ SAD (or RMSE) is used to evaluate the performance of estimating fluorescence spectra and concentrations.

In this synthetic experiment, for all algorithms the parameter settings are as follows: an unimodality of spectra is introduced in 1th and 2nd endmember for MCR-ALS; the penalty coefficient of sparsity constraint is 0.01 for NMF${{l}_{1}}$; the sparsity of concentration distribution is 0.65 at NMFsc; the sparsity value for both the first and second concentrations is 0.73 at sNMF. The number of endmember for all algorithms is 3.

**Fig. 2:**
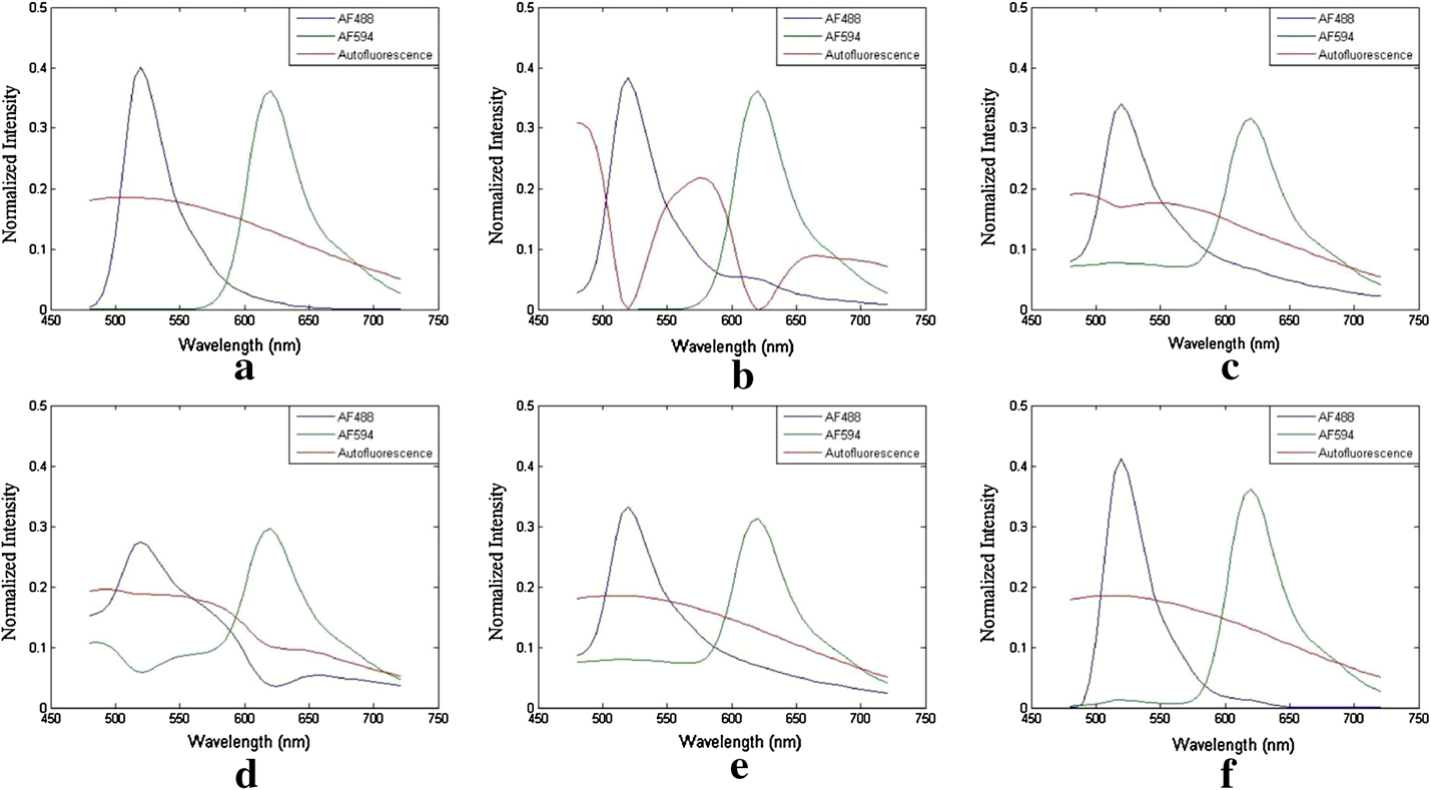
Ground truth synthetic data when SNR=30 and the different unmixed spectra of AF488, AF594 and AF obtained with different algorithms. **a** Ground truth of fluorescence spectra for two simulated fluorescent targets and one simulated AF. The different unmixed spectra of AF488, AF594 and AF obtained with all algorithms: **b** MCR-ALS, **c** NMF${{l}_{1}}$, **d** NMFsc, **e** sNMF, **f** thNMF

Figure 2 shows the ground truth and unmixed fluorescence spectra of synthetic data when SNR = 30 dB. The unmixed AF spectrum for MCR-ALS in Fig. 2b is totally wrong and the unmixed spectrum of AF488 is somewhat dissimilar to the ground truth (Fig. 2a). All unmixed fluorescence spectra of NMFsc in Fig. 2d have large artifacts. The fluorescence spectra of AF488 and AF594 for NMF${{l}_{1}}$ in Fig. 2c and for sNMF in Fig. 2e have abnormal offsets and distortions between 480 and 650 nm. All unmixed endmembers by our thNMF method (Fig. 2f) obtain the best fluorescence spectra that are most similar to the ground truth of spectra.

**Fig. 3:**
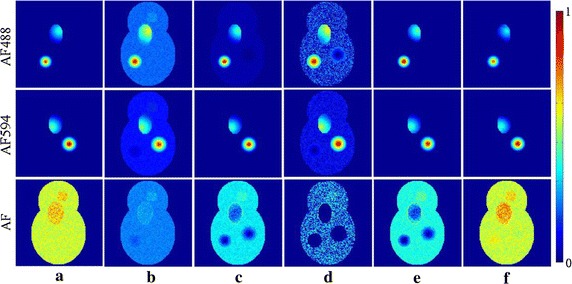
Ground truth synthetic data when SNR = 30 and the different unmixed concentrations obtained with different algorithms. The three rows correspond to two target fluorescence endmembers: AF488 and AF594, and the background AF. **a** Ground truth of fluorescence concentrations for simulated AF488, AF594 and AF. The different unmixed abundances of AF488, AF594 and AF obtained with: **b** MCR-ALS, **c** NMF${{l}_{1}}$, **d** NMFsc, **e** sNMF, **f** thNMF

**Fig. 4:**
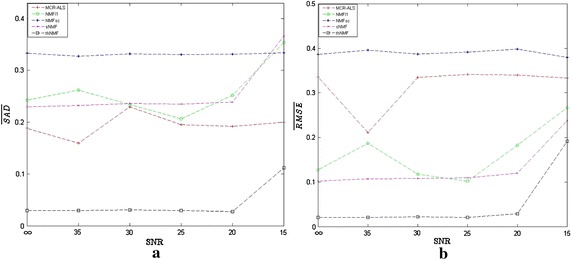
Evaluation for unmixing results of synthetic data. **a** $\overline{SAD}$ and **b** $\overline{RMSE}$ as functions of SNRs

The normalized unmixing results of all endmembers obtained with all algorithms are displayed in Fig. 3, where the unit of the color bar is normalized from 0 (zero intensity) to 1 (the maximum intensity of the final unmixing results obtained with all methods). The unmixed AF488 and AF594 for MCR-ALS in Fig. 3b and NMFsc in Fig. 3d are not localized in spatial distributions, such that they are very dissimilar to the ground truth of simulated AF488 and AF594 fluorescences in Fig. 3a. There are obvious holes in the spatial distributions of unmixed AF by NMF${{l}_{1}}$ in Fig. 3c, NMFsc in Fig. 3d and sNMF in Fig. 3e. Moreover, from the color scale representations of unmixing results, we can see that the concentrations of AF488 and AF594 for MCR-ALS, NMF${{l}_{1}}$, NMFsc and sNMF are much larger than the ground truth value of concentrations in Fig. 3a. Though sNMF can get desirable sparse spatial distribution for AF488 and AF594, the unmixed concentrations are larger than the ground truth concentrations of these two fluorescence targets; besides, the unmixed AF have two holes in the locations of fluorescence targets. Compared with other methods, only our thNMF method in Fig. 3f achieves the best unmixing results that are very similar to the ground truth of simulated data.

Figure 4 manifests the mean values of SAD and RMSE as functions of the SNRs ranging from $\infty$ (without any noise) to 15 dB in steps of 5 dB for three endmembers. We can see that the NMFsc obtains the worst results for both the SAD and the RMSE. Meanwhile, the proposed thNMF has not only the best mean SAD but also the smallest mean RMSE to demonstrate its robustness against noise corruption.

### Experimental results with real fluorescent data

In this section, we apply thNMF algorithm to in-vivo fluorescent data acquired with a Bio-Real Quick View 3000 imaging system (Bio-Real Sciences, Austria), which is an epi-illumination planar imaging system equipped with electron multiplying charge coupled device (EMCCD) and 150 watt Xenon arc lamp as a light source. The EMCCD (Andor Technology, Ireland) has high quantum efficiency in the range of 400–950 nm which is our interest of fluorescence imaging. In the epi-illumination fluorescence imaging system, the EMCCD gets the emission light through the multiple bandpass (30 nm) emission filters covering 525, 542, 579, 624 and 716 nm (Semrock, Rochester, USA).

In the following three in-vivo experiments, we use one nude and two BALB/c mice with subcutaneous specific fluorescent dyes to confirm the effectivity of thNMF for preclinical applications. This subcutaneous injection of dye is sometimes adopted in clinical fluorescence unmixing researches [2–4] and can easily spread the dye through mouse’s body. The animal experiments were approved by our institutional review board. The nude mouse has weak AF and is often used in fluorescence imaging, while the BALB/c mouse has much stronger AF than nude mouse due to the fur of BALB/c mouse being easy to be excited. Furthermore, two fluorescent dye pairs AF488/AF594 and AF488/AF555 are used as fluorescent markers, in which the AF488/AF555 pair has more spectral overlap between two dyes’s spectra than the AF488/AF594 has (see the following sections for detail). Both of these multiple-target fluorescence emissions have spectral overlap with the spectrum of complex background AF. To reduce the AF in stomach region, the mouse may be fed with alfalfa-free diet in preclinical applications. However, the mice are normally fed before in-vivo experiments in this work. To demonstrate the raw images and unmixing results clearly, all the observations and unmixing results are displayed in pseudo-color mapping based on logarithmic scaling (or linear scaling) and overlaid with the corresponding gray-scale photographic image of mouse.

We demonstrate four classic spectral unmixing algorithms (MCR-ALS, NMF${{l}_{1}}$, NMFsc and sNMF mentioned in the above section) for comparison purpose. All algorithms assume that there are three endmembers for spectral unmixing, or *K*=3. The parameter settings for all algorithm are as follows: an unimodality constraint of spectra is introduced in the first and second endmember for MCR-ALS; the penalty coefficient of sparsity constraint is 0.001 for NMF${{l}_{1}}$; the sparsity of concentrations is 0.88 in NMFsc; the sparsity value for both the first and second concentrations is 0.87 for sNMF.

Unlike the in-vitro fluorescence, the photons from the in-vivo target fluorophores are located inside tissues and may be greatly attenuated before reaching the mouse surface due to light scattering and absorption. This attenuation varies with fluorescent dye’s injection depth, tissue type, and wavelength, it is hence impossible to obtain the true concentrations of all endmembers in in-vivo multiple target fluorophores. Besides, the true spectrum of AF is unknown in the real in-vivo experiments. Therefore, we use SAD to evaluate the spectrum estimation of endmember AF488 and AF594. Besides, the visual inspection is still a useful assessment method to evaluate algorithm’s performance. We also introduce a sparsity value $\phi$ of column vector of concentration matrix $\mathbf {C}$ [27] to evaluate the concentration estimation. In the real experiments, the sparsity values of fluorescent dyes $\mathbf {C}_F$ must have relatively large values because of the spatial sparseness of fluorescence targets while the sparsity value of AF $\mathbf {C}_A$ must have a relatively small value because of the dispersive distribution of AF.

#### The nude mouse

**Fig. 5:**
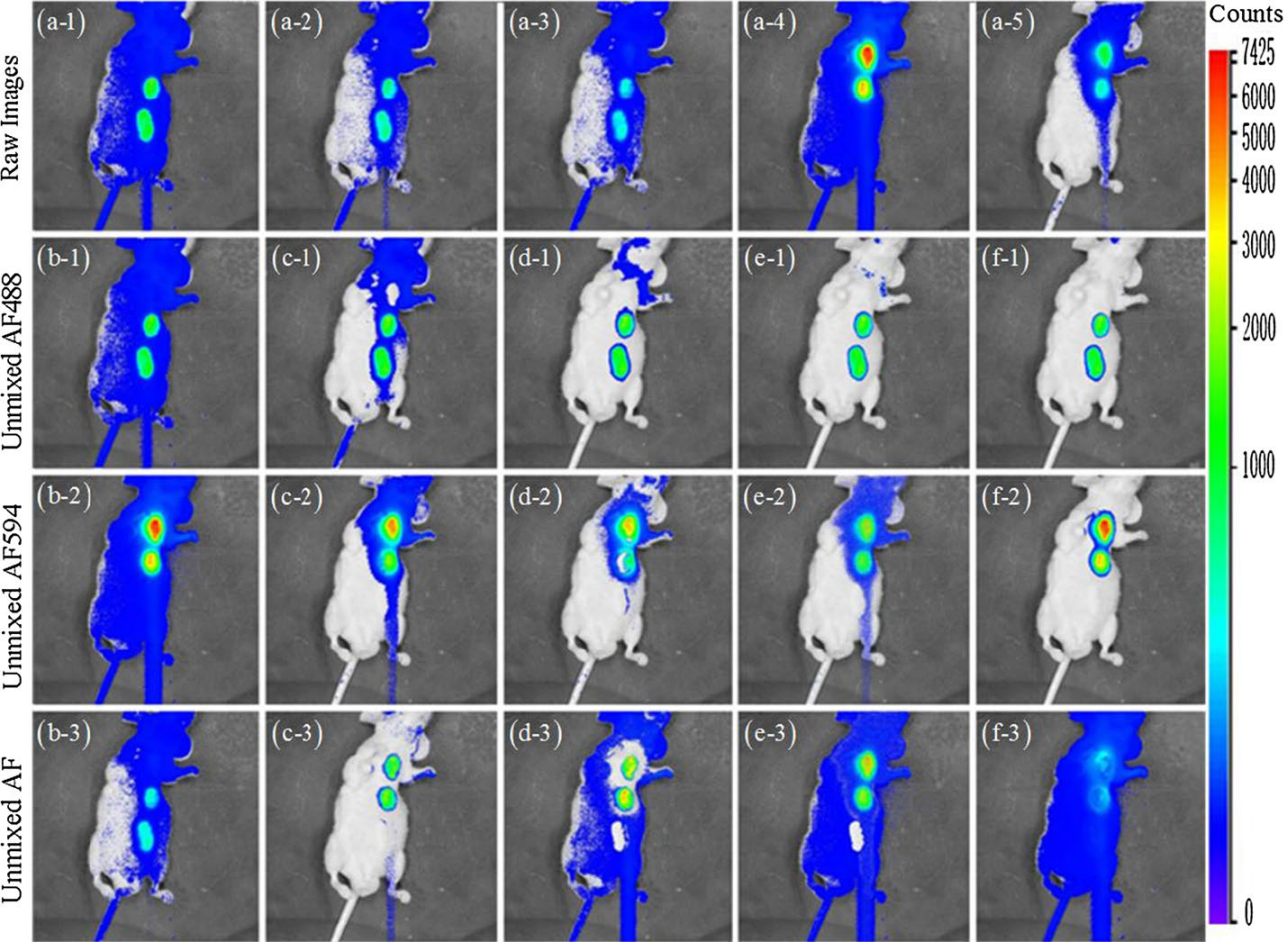
Unmixing results for nude mouse experiment. **a-1**–**a-5** Raw fluorescence images acquired at the 525, 542, 579, 624 and 716 nm emission filters after subcutaneous injection of AF488 and AF594 dyes into nude mouse. The AF488 in the first three images is excited at 474 nm, the AF594 in the last two images is excited at 565 nm. The unmixed AF488, AF594, and AF obtained with: **b-1**–**b-3** MCR-ALS, **c-1**–**c-3** NMF${{l}_{1}}$, **d-1**–**d-3** NMFsc, **e-1**–**e-3** sNMF, (f-1–(f-3) thNMF

In the first two experiments, the AF488 and AF594 fluorescent dyes are diluted to 0.1 μg ml$^{-1}$. The raw images are sparsely acquired through five emission glass filters from 525 to 716 nm. The first three images are excited at 474 nm, the last two images are excited at 565 nm. In the first nude mouse experiment, AF488 is injected at the bottom of the body with 20 ng dye while AF596 near the neck with the same quantity, and a mixture of each dye with 10 ng is located at the middle of the body.

Figure 5(a-1)–(a-5) display the raw spectral images acquired from five emission filters. The pseudo-color mapping is based on logarithmic scaling to clearly display the detailed change of weak AF in all spectral channels. The AF488 is displayed on the channels 525, 542 and 579 nm while the AF594 on the 624, 716 nm. It seems that there is almost no cross-talk between these two fluorescent dyes. However, the AF is weakly presented on the background surface of mouse and overlapped with AF488 and AF594 in all multispectral observations. Therefore, this nude mouse experiment is appropriate to test the unmixing performance in removing weak AF from the multiple fluorescence targets.

Five algorithms’ unmixing results for in-vivo fluorescence imaging are displayed in Fig. 5(b-1)–(b-3) for MCR-ALS, Fig. 5(c-1)–(c-3) for NMF${{l}_{1}}$, Fig. 5(d-1)–(d-3) for NMFsc, Fig. 5(e-1)–(e-3) for sNMF, Fig. 5(f-1)–(f-3) for our thNMF. MCR-ALS and NMF${{l}_{1}}$ methods still overlap the fluorescent dyes with background AF. Though the unmixed fluorescent dyes obtained with NMFsc and sNMF are separated to some degree, the unmixing is not complete and the fluorescent dyes with their strong intensities are still overlapped with the AF in Fig. 5(d-3) and Fig. 5(e-3). Only the proposed thNMF can successfully separate multiple fluorescent dyes and AF.

**Table 1:** SAD results on the nude mouse data

	MCR-ALS	NMF${{l}_{1}}$	NMFsc	sNMF	thNMF
AF488	0.3742	0.1536	0.1569	0.1495	*0.1479*
AF594	*0.0876*	0.1385	0.1518	0.1116	*0.0903*
Mean	0.2309	0.1460	0.1544	0.1305	*0.1191*

**Table 2:** Sparsity values on the nude mouse data

	MCR-ALS	NMF${{l}_{1}}$	NMFsc	sNMF	thNMF
AF488	0.8372	0.8630	0.8900	0.8900	0.8946
AF594	0.8667	0.8761	0.8900	0.8899	0.8995
AF	0.8077	0.9076	0.8900	0.8709	*0.6138*

Table 1 quantifies all algorithms’ unmixing performances using the SAD similarity, while Table 2 represents the sparsity values of concentrations for all endmembers. Table 1 shows that the unmixing results of thNMF get the smallest value of SAD for the AF488 endmember and the smallest value of mean SAD. The proposed thNMF also obtains the second smallest value of SAD for the AF594. Though MCR-ALS gets the smallest value of SAD for the AF594 endmember, the sparsity value 0.8077 of concentration for AF achieved with MCR-ALS is larger than the sparsity value 0.6138 achieved with thNMF, which is in conflict with the reality that the sparsity value of fluorescent dye is much larger than that of AF. Considering these factors comprehensively, we confirm that the proposed thNMF gets the best unmixing results with fully removing the AF for the nude mouse experiment.

#### The BALB/C mouse 1

**Fig. 6:**
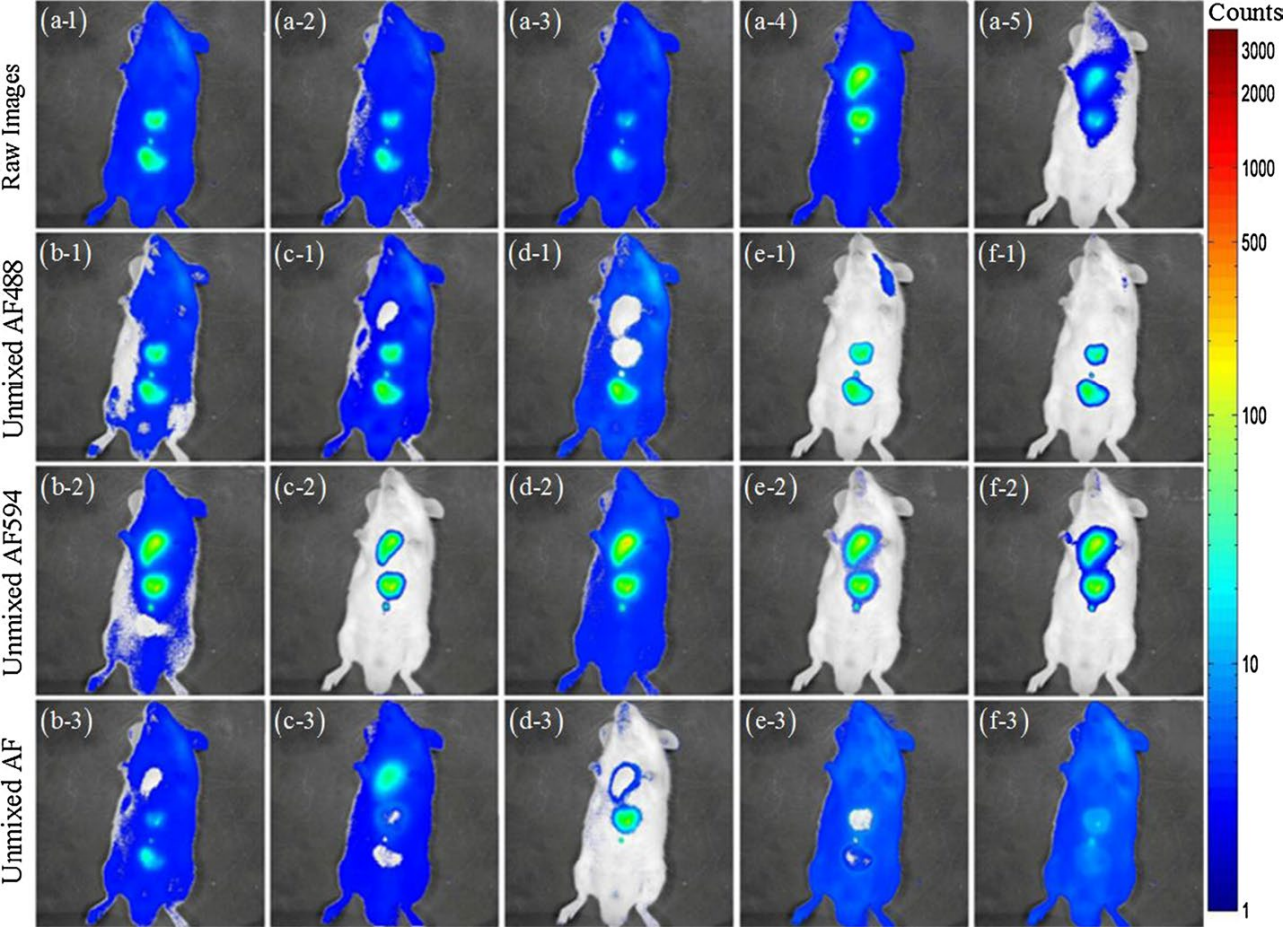
Unmixing results for BALB/c mouse experiment 1. **a-1**–**a-5**. Raw fluorescence images acquired at the 525, 542, 579, 624 and 716 nm emission filters after subcutaneous injection of AF488 and AF594 dyes into BALB/c mouse. The AF488 in the first three images is excited at 474 nm, the AF594 in the last two images is excited at 565 nm. The unmixed AF488, AF594, and AF obtained with: **b-1**–**b-3** MCR-ALS, **c-1**–**c-3** NMF${{l}_{1}}$, **d-1**–**d-3** NMFsc, **e-1**–**e-3** sNMF, **f-1**–**f-3** thNMF

The experimental condition for BALB/c mouse is similar to previous nude mouse experiment. Fig. 6(a-1)–(a-5) show the raw spectral images acquired from five emission filters. The AF is apparently overlapped with the AF488 at the first three channels and the AF594 at the last two channels, respectively. The BALB/C mouse introduces more AF and more attenuation of fluorescence signal than the nude mouse dose due to BALB/C mouse hair, which further lowers the contrast between the target fluorescence and background AF in BALB/C mouse compared to the nude mouse. The AF distribution is more irregular and large than that of nude mouse. Moreover, BALB/C mouse’s AF intensity is much stronger than nude mouse’s AF intensity. Especially, the AF at the upper right part of BALB/C mouse has an intensity comparable to the AF488 intensity at the channels of 525, 542 and 579 nm.

Five algorithms’ unmixing results are illustrated in Fig. 6(b-1)–(b-3) for MCR-ALS, Fig. 6(c-1)–(c-3) for NMF${{l}_{1}}$, Fig. 6(d-1)–(d-3) for NMFsc, Fig. 6(e-1)–(e-3) for sNMF, Fig. 6(f-1)–(f-3) for our thNMF. The unmixed fluorescent dyes obtained with MCR-ALS, NMF${{l}_{1}}$ and NMFsc are still overlapped with background AF, while the unmixed fluorescent dyes obtained with NMFsc in Fig. 6(d-1)–(d-3) are still overlapped at different locations. Though the unmixing results of sNMF (Fig. 6(e-1)–(e-3)) are similar to thNMF’s results (Fig. 6(f-1)–(f-3)), the concentration distribution of AF is affected by the fluorescent dyes in the middle of body such that there is an obvious hole in AF at the bottom of body in Fig. 6(e-3). Among all unmixing results from all five algorithms, the unmixing result of thNMF is best and the cross-talk between AF and the two fluorescent dyes is reduced to a negligible level.

**Table 3:** SAD Results on the BALB/c mouse data

	MCR-ALS	NMF${{l}_{1}}$	NMFsc	sNMF	thNMF
AF488	0.2670	0.2889	0.2748	0.1881	*0.1482*
AF594	0.1212	0.1533	0.1260	0.1202	0.1204
Mean	0.1941	0.2211	0.2004	0.1542	*0.1343*

Table 3 quantifies the different fluorescence unmixing performances using the SAD similarity, while Table 4 represents the sparsity values of concentrations for all endmembers. The evaluation results in Table 3 are similar to those of nude mouse experiment. Our thNMF obtains the smallest mean SAD, the smallest SAD value of AF488 and the second smallest SAD value of AF594, while sNMF gets the smallest SAD value of AF594 endmember. All SAD values of AF594 endmember are close to each other for all algorithms except NMF${{l}_{1}}$ algorithm.

**Table 4:** Sparsity values on the BALB/c mouse 1 data

	MCR-ALS	NMF${{l}_{1}}$	NMFsc	sNMF	thNMF
AF488	0.3848	0.3656	0.7000	0.8734	0.8855
AF594	0.7180	0.8821	0.7000	0.8677	0.8621
AF	0.2939	0.4122	0.7000	0.1397	*0.1176*

Table 4 displays that the sparsity value of AF for NMF${{l}_{1}}$ is larger than that of AF488. Besides, the sparsity value of AF for NMFsc is equal to that of AF488 (or AF594). These sparsity results simply do not conform to the reality that the sparsity value of AF is much smaller than that of fluorescent dye. However, thNMF and sNMF can achieve good sparsity value for all concentrations in Table 4. More precisely, thNMF has better sparsity values of AF488 and AF than sNMF dose. Taking all these factors into account, we can find that thNMF can obtain the best unmixed result as compared with other four algorithms in the experiment of BALB/c mouse.

#### The BALB/C mouse 2

**Fig. 7:**
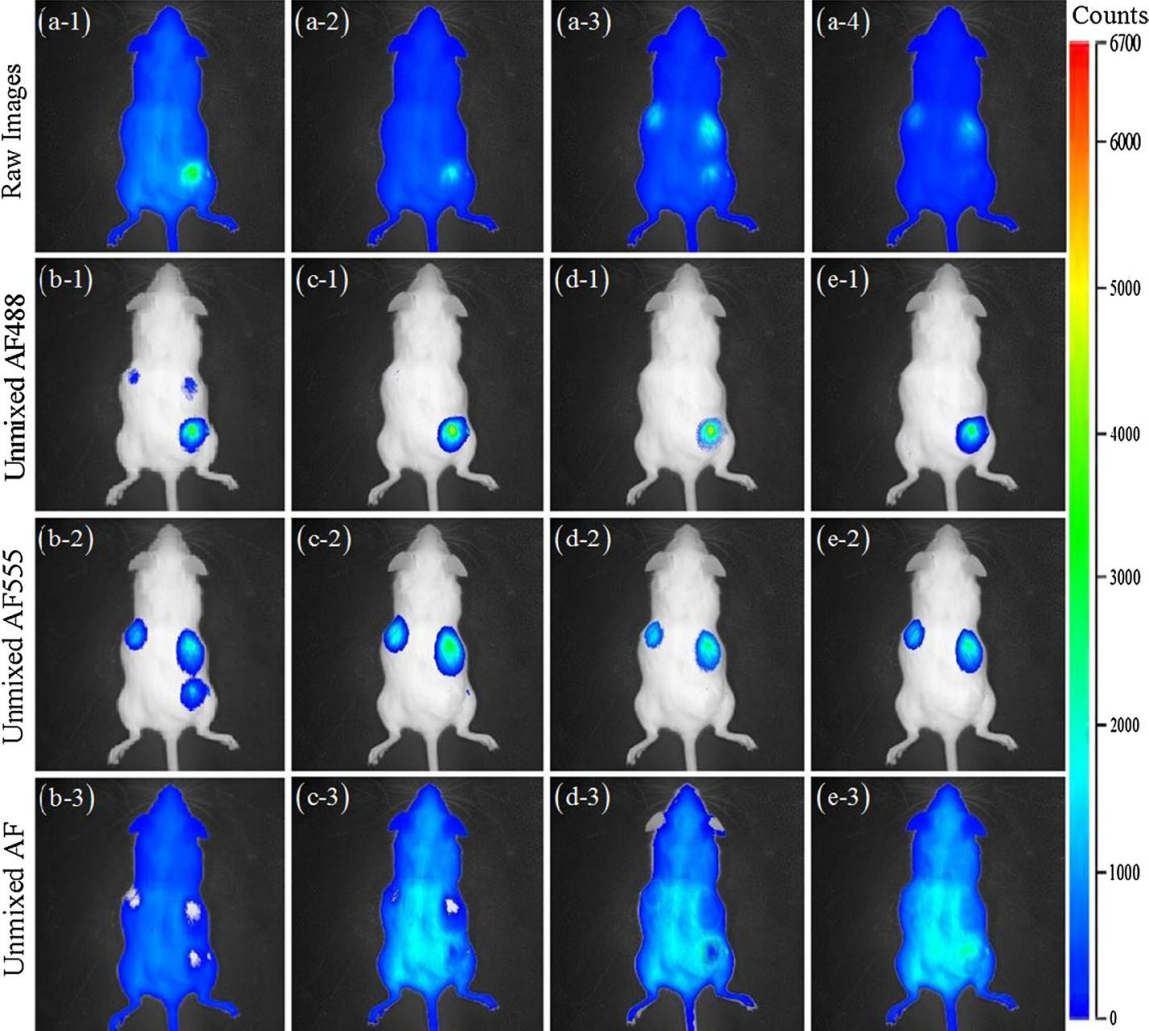
Unmixing results for BALB/c mouse experiment 2. **a-1**–**a-4** Raw fluorescence images acquired at the 525, 542, 579 and 624 nm emission filters after subcutaneous injection of AF488 and AF555 dyes into BALB/c mouse. The AF488 in the first three images is excited at 474 nm and AF555 in the last two images at 500 nm. The unmixed AF488, AF555, and AF obtained with: **b-1**–**b-3** MCR-ALS, **c-1**–**c-3** NMF${{l}_{1}}$, **d-1**–**d-3** sNMF, **e-1**–**e-3** thNMF

In the last experiment, the AF488 and AF555 used as two target fluorophores are diluted to 0.1 μg ml$^{-1}$. AF488 (50 ng) is subcutaneously injected at the bottom right of the mouse body and AF555 (50 ng) on the left and right sides of the middle part of body. Fig. 7(a-1)–(a-4) show raw fluorescence images that are sparsely acquired at the 525, 542, 579, 624 nm emission filters. Using linear scaling for the pseudo-color mapping can clearly demonstrate the detailed change of strong AF, which has clear spectral overlap with AF488 and AF555 in all spectral channels. The AF488 is excited at 474 nm and displayed nearly on all the four channels while the AF555 is excited at 500 nm and emitted on the 579 and 624 nm channels. In addition to the strong AF overlapping greatly with the AF488 and AF555, there is also distinct cross-talk between these two target fluorophores.

We compare the proposed method to MCR-ALS, NMF${{l}_{1}}$ and sNMF that have good performances in previous experiments. The unmixing results are illustrated in Fig. 7(b-1)–(b-3) for MCR-ALS, Fig. 7(c-1)–(c-3) for NMF${{l}_{1}}$, Fig. 7(d-1)–(d-3) for sNMF, Fig. 7(e-1)–(e-3) for thNMF. Except for the MCR-ALS, the other three methods, i.e. NMF${{l}_{1}}$, sNMF and thNMF, can separate the fluorescent targets in Fig. 7(c-1)–(c-2), (d-1)–(d-2), (e-1)–(e-2) from AF, respectively. But there are missing parts in AF component in Fig. 7(c-3) for NMF${{l}_{1}}$ and Fig. 7(d-3) for sNMF. Though the sparse acquisition from only four spectral channels exacerbates the ill-posed problem of spectral unmixing, the proposed thNMF can fully remove the intense AF from the two target fluorophores and obtain best unmixing results among all algorithms. Besides these visual assessment, Table 5 confirms that the proposed algorithm achieves the smallest values of average SAD.

**Table 5:** SAD Results on the BALB/c mouse 2 Data

	MCR-ALS	NMF${{l}_{1}}$	sNMF	thNMF
AF488	0.2161	0.1567	0.1173	*0.0462*
AF555	0.2481	0.2375	0.2134	*0.2053*
Mean	0.2321	0.1971	0.1654	*0.1258*

## Discussion and conclusions

By spatially distinguishing the target fluorophores from background AF within equality constrained [19] HALS framework, we have proposed thNMF algorithm for in-vivo multispectral fluorescence imaging to divide the whole update process of concentration matrix $\mathbf {C}$ into two relevant sub-processes, at which the concentrations of target fluorophores and background AF are separately updated with a spatial sparsity constraint being solely imposed on the target fluorophore. A detailed comparison with other four state-of-arts spectral unmixing algorithms is conducted to confirm the excellent performance of thNMF algorithm. In NMF-based spectral unmixing, the initial estimates of the endmembers and AF are important for solving the ill-posed problem of NMF. As in [4], all the methods for comparison in this work use calibration spectra to initialize the spectral matrix $\mathbf {S}$. This initialization alone can not guarantee NMF’s convergence to an ideal solution. Furthermore, the very sparse acquisition of multispectral fluorescence imaging has incomplete measurement and severe discontinuity problems over multispectral fluorescence emissions with various complex AF, such that it aggravates the ill-posed nature of the inverse problem of spectral unmixing. Under such sparse acquisition, the unimodality of spectra constraint (for MCR-ALS) and other sparsity constraints for some states-of-arts NMF-based spectral unmixing algorithms can not facilitate achieving the optimal unmixed solutions. However, by employing equality constraint along with partial sparsity constraint during HALS optimization where fixing background AF (resp. sparsity constrained target fluorophores) can make the update of sparsity constrained target fluorophores (resp. background AF) as accurate as possible, the thNMF algorithm can successfully tackle the ill-posed and sparse acquisition problems in the NMF framework according to the different spatial distribution of the background AF and the target fluorophores.

Considering the different spatial characteristics of endmembers from different hierarchies in the in-vivo fluorescence imaging, the proposed thNMF algorithm can easily add different spatial regularization constraints into different hierarchies to get optimal solution. Although we concentrate on sparse multispectral imaging and fluorescence unmixing, two-hierarchical updating that distinguishes the target from background signal can be an interesting inspiration for research of multispectral fluorescence imaging and blind source separation. This is because most multi-modality signals can be decomposed into two hierarchies: one hierarchy of localized signals of interest with its sparse representation and another hierarchy of background noisy signal with its widely distributed spatial existence.

## Abbreviations

NMF: nonnegative matrix factorization
SUM: spectral unmixing (SUM)
thNMF: two-hierarchical NMF
HALS: hierarchical alternating least squares
ANLS: alternating nonnegative least squares.
